# Genes associated with Type 2 Diabetes and vascular complications

**DOI:** 10.18632/aging.101375

**Published:** 2018-02-04

**Authors:** Alberto Montesanto, Anna Rita Bonfigli, Paolina Crocco, Paolo Garagnani, Maria De Luca, Massimo Boemi, Elena Marasco, Chiara Pirazzini, Cristina Giuliani, Claudio Franceschi, Giuseppe Passarino, Roberto Testa, Fabiola Olivieri, Giuseppina Rose

**Affiliations:** 1 Department of Biology, Ecology and Earth Sciences, University of Calabria, Rende, Italy; 2 Scientific Direction, INRCA-IRCCS National Institute, Ancona, Italy; 3 Department of Experimental, Diagnostic and Specialty Medicine (DIMES), Alma Mater Studiorum, University of Bologna, Bologna, Italy; 4 Clinical Chemistry, Department of Laboratory Medicine, Karolinska Institutet at Huddinge University Hospital, Stockholm, Sweden; 5 Department of Nutrition Sciences, University of Alabama at Birmingham, Birmingham, AL35294, USA; 6 Diabetology Unit, INRCA-IRCCS National Institute, Ancona, Italy; 7 IRCCS Istituto delle Scienze Neurologiche di Bologna, Bologna, Italy and Department of Experimental, Diagnostic and Specialty Medicine, Alma Mater Studiorum-University of Bologna, Bologna, Italy; 8 Clinical Laboratory and Molecular Diagnostics, INRCA-IRCCS National Institute, Ancona, Italy; 9 Department of Clinical and Molecular Sciences, DISCLIMO, Università Politecnica delle Marche, Ancona, Italy; 10 Center of Clinical Pathology and Innovative Therapy, National Institute INRCA-IRCCS, Ancona, Italy; * Equal contribution

**Keywords:** Type 2 Diabetes, diabetes complications, genetic profile, SNP, aging

## Abstract

Type 2 Diabetes (T2D) is a chronic disease associated with a number of micro- and macrovascular complications that increase the morbidity and mortality of patients. The risk of diabetic complications has a strong genetic component. To this end, we sought to evaluate the association of 40 single nucleotide polymorphisms (SNPs) in 21 candidate genes with T2D and its vascular complications in 503 T2D patients and 580 healthy controls. The genes were chosen because previously reported to be associated with T2D complications and/or with the aging process. We replicated the association of T2D risk with I*GF2BP* rs4402960 and detected novel associations with *TERT* rs2735940 and rs2736098. The addition of these SNPs to a model including traditional risk factors slightly improved risk prediction. After stratification of patients according to the presence/absence of vascular complications, we found significant associations of variants in the *CAT*, *FTO*, and *UCP1* genes with diabetic retinopathy and nephropathy. Additionally, a variant in the *ADIPOQ* gene was found associated with macrovascular complications. Notably, these genes are involved in some way in mitochondrial biology and reactive oxygen species regulation. Hence, our findings strongly suggest a potential link between mitochondrial oxidative homeostasis and individual predisposition to diabetic vascular complications.

## Introduction

Over the last three decades, the incidence of T2D and related complications has rapidly increased worldwide [1]. T2D is a complex metabolic disorder that is caused by multiple factors [2]. Classic genetic analyses performed in family and twin studies have clearly shown that up to 70% of the variance in T2D susceptibility in the population is explained by genetic factors [3]. Individuals with T2D-affected siblings have a two-three fold increased risk of developing T2D compared with the general population [4]. Additionally, we previously showed that the frequencies of T2D risk variants are inversely correlated with a decreasing health trend and a reduced chance to becoming a centenarian [5]. In recent years, genome-wide association studies (GWAS) based on the common disease-common variant hypothesis have fully confirmed the polygenic nature of T2D and identified approximately 100 T2D susceptibility SNPs [6–8]. However, these variants only capture approximately 10% of the heritable risk for T2D [9]; hence, much of the heritability is still to be discovered. Aggregating information from multiple risk SNPs (each with a small effect) into a genetic risk profile has become widely used in epidemiologic studies [10]. This approach is suitable not only for improving performance in predicting overall disease risk, but also for developing population-based screening and prevention programs [10]. Nonetheless, when SNPs associated with increased T2D risk were integrated into risk models that included both conventional and genetic risk factors, only little improvement was obtained in the predictive performance of genotype information [11].

T2D related complications, such as micro- and macroangiopathy, appear to be strongly interconnected, with microvascular diseases promoting macrovascular complications [1]. Micro- and macrovascular complications are associated with long-term damage and failure of a number of organ systems [1]. Recently, much attention has been focused on the management of macrovascular complications, such as stroke and acute coronary syndromes. However, microangiopathy is also associated with increased damage in different organs, thus promoting retinopathy, nephropathy and neuropathy [12–15]. It is well established that the risk of diabetic complications has a strong genetic component; however, only a handful number of genetic loci have been so far discovered that influence individual predisposition to T2D complications [16]. Considering the high morbidity and mortality associated with T2D complications [17], there is a strong need to detect genetic risk factors for T2D complications. Moreover, the identification of novel candidate genes associated with T2D vascular complications will further contribute to our understanding of the mechanisms underlying disease progression.

In this study, we genotyped 40 SNPs in 21 candidate genes for association with risk of T2D and its vascular complications in 1083 subjects previously recruited to be a part of a case-control study [18]. This set of SNPs was carefully selected among: i) SNPs previously found associated with T2D by GWAS; ii) SNPs of genes associated with micro- and macrovascular T2D complications; iii) SNPs previously associated with metabolic diseases; and iv) SNPs of genes considered to be relevant for telomere stability and to contribute to the aging process. This final set of candidate genes was chosen because age is an important risk factor for T2D. We reasoned that genetic variants associated with the intrinsic processes of aging might have a role in the progression to diabetic complications. The first objective of this study was to perform basic association analysis for all individual SNPs with T2D risk. This was followed by the generation of a genetic profile to evaluate whether the inclusion of the genetic variants would enhance the predictive power of a model including traditional risk factors. After stratification of patients according to the presence/absence of vascular complications, the final objective was to test for association between each SNP and risk of T2D complications.

## RESULTS

## SNPs association with T2D

We analysed a cohort of Italian subjects including 503 diabetic patients and 580 non-diabetic controls. After the quality control (QC) sample phase, we removed 143 subjects because their genetic profiles were not complete (< 90%). The final sample count comprised 940 recruits (460 males and 498 females) of which 435 were cases and the remaining 505 controls. At the SNP level, the QC phase excluded a total of 10 SNPs. In particular, six SNPs were excluded due to missing frequency (MiF) data higher than 10% per locus (rs669173, rs2853669, rs4880, rs13266634, rs7901695, rs8047395), two SNPs were excluded because they showed a significant deviation from Hardy-Weinberg equilibrium (HWE) in control subjects (rs147057, rs16889462), and two SNPs were excluded because their minor allele frequency (MAF) was lower than 5% (rs2237892, rs3811791). The final dataset included 30 high quality SNPs that were tested for association with T2D and its complications.

The demographic, clinical, and anthropometric characteristics of the analysed cohort are presented in Table 1. Affected individuals were older (mean age 65.71±7.9 *vs* 58.59±12.2 years) and with a higher proportion of males with respect to the control group (56.6% *vs* 41.2%). Moreover, significant differences in several quantitative metabolic phenotypes were observed between T2D patients and controls (Table 1).

**Table 1:** Demographic and clinical characteristics of diabetic and non-diabetic participants.

**Variable**	**Cases (N=435)**	**Controls (N=505)**	**P-value**
Age (mean, SD)	65.71 (7.90)	58.59 (12.23)	<0.001
Gender (males, %)	246 (56.6%)	208 (41.2%)	<0.001
BMI, kg/m^2^ (mean, SD)	28.7 (4.59)	27.1 (5.04)	<0.001
Family history (n, %)	314 (72.5)	105 (21.3)	<0.001
WHR (mean, SD)	0.93 (0.08)	0.87 (0.11)	<0.001
HOMA-IR (mean, SD)	2.91 (2.79)	1.58 (1.48)	<0.001
Fasting Glucose, mg/dl (mean, SD)	162.82 (47.50)	92.98 (11.65)	<0.001
Insulin, µIU/mL (mean, SD)	7.07 (5.39)	6.71 (5.51)	0.324
HbA1c, % (mean, SD)	7.44 (1.27)	5.64 (0.39)	<0.001
Retinopathy (n, %)	111 (25.5%)	0 (0.0%)	<0.001
Nephropathy (n, %)	54 (12.4%)	0 (0.0%)	<0.001
Neuropathy (n, %)	76 (17.5%)	0 (0.0%)	<0.001
Ischemic heart disease and stroke [(n, %)]	76 (17.5%)	0 (0.0%)	<0.001
Telomere length (mean, SD)	0.43 (0.20)	0.48 (0.18)	<0.001

Abbreviations: SD, standard deviation; BMI, Body Mass Index; WHR, waist-to-hip ratio; HOMA-IR, homeostasis model assessment of insulin resistance; HbA1c, glycosylated haemoglobin.

Table 2 summarizes the results obtained from association analyses. After adjustments for age, gender, BMI, and familiarity, the most significant results involved *IGF2BP2* (rs4402960) and *TERT* (rs2735940 and rs2736098). In particular, *IGF2BP2* rs4402960 T-allele and *TERT* rs2735940-C allele were associated with T2D with an odds ratio (OR) of 1.28 (95% CI 1.01-1.62; p=0.038) and 1.34 (95% CI 1.05-1.71; p=0.017) per risk allele, respectively. On the contrary, the *TERT* rs2736098-A allele conferred a protective effect against T2D with an OR of 0.64 (95% CI 0.49-0.84; p=0.001). Because of the pattern of linkage disequilibrium (LD) (r^2^=0.47), we also found a borderline association with *TERT* rs2736109 (OR= 0.79, 95% CI 0.62-1.01, p=0.061).

**Table 2:** Association analysis of selected SNPs with Type 2 Diabetes.

**Gene**	**SNP ID**	**Major/Minor allele**	**MAF**	**OR (95% CI)^a^**	**P-value**
ADIPOQ	rs266729	C/G	0.239	1.08 (0.83-1.42)	0.555
ADIPOQ-AS1	rs1063539	G/C	0.111	1.03 (0.71-1.48)	0.889
APOE	rs440446	G/C	0.375	1.16 (0.91-1.48)	0.224
CAT	rs1001179	G/A	0.214	0.93 (0.7-1.24)	0.629
CLPTM1L	rs401681	C/T	0.40	1.09 (0.86-1.39)	0.467
DDAH1	rs13373844	A/C	0.334	1.09 (0.85-1.40)	0.496
DDAH1	rs7521189	A/G	0.466	0.98 (0.60-1.57)	0.840
EPO	rs1617640	T/G	0.330	1.08 (0.84-1.39)	0.544
EPO	rs507392	T/C	0.334	1.13 (0.88-1.46)	0.339
EPO	rs551238	A/C	0.329	1.1 (0.86-1.42)	0.435
FTO	rs1121980	C/T	0.477	1.01 (0.8-1.27)	0.928
FTO	rs1421085	T/C	0.464	1.01 (0.8-1.27)	0.933
FTO	rs17817449	T/G	0.446	1.06 (0.84-1.33)	0.621
FTO	rs1800592	A/G	0.249	0.85 (0.65-1.11)	0.221
FTO	rs8050136	C/A	0.447	1.02 (0.81-1.29)	0.847
FTO	rs9939609	T/A	0.441	1.03 (0.82-1.3)	0.804
HIF1A	rs11549465	C/T	0.151	1.08 (0.78-1.49)	0.651
IGF2BP2	rs4402960	G/T	0.347	**1.28 (1.01-1.62)**	**0.038**
IRS1 (500 kb downstream)	rs2943641	C/T	0.384	1.11 (0.87-1.40)	0.404
KCNJ11	rs5215	T/C	0.341	1.01 (0.79-1.28)	0.963
TCF7L2	rs7903146	C/T	0.405	1.22 (0.96-1.55)	0.097
TERC	rs12696304	C/G	0.266	1.06 (0.82-1.36)	0.680
TERT	rs2735940	T/C	0.415	**1.34 (1.05-1.71)**	**0.017**
TERT	rs2736098	G/A	0.303	**0.64 (0.49-0.84)**	**0.001**
TERT	rs2736109	G/A	0.423	0.79 (0.62-1.01)	0.061
UCP1	rs3811787	T/G	0.255	0.85 (0.65-1.11)	0.225
UCP1	rs45539933	C/T	0.063	0.99 (0.61-1.6)	0.969
UCP2	rs660339	C/T	0.354	0.95 (0.74-1.21)	0.676
UCP3	rs1800849	C/T	0.15	0.87 (0.62-1.21)	0.404
VEGFA	rs3025021	C/T	0.349	0.87 (0.67-1.13)	0.285

Abbreviations: MAF, Minor Allele Frequency; OR, odds ratio; CI, confidence interval.

Significant p-values (<0.05) are highlighted in bold.

^a^ For each SNP an adjusted additive model was considered.

## Genetic risk profile

Table 3 reports the results of the stepwise regression procedure, adjusted for age, gender, BMI and familiarity, performed to evaluate the combined effect of genetic variables on T2D susceptibility. This analysis confirmed rs4402960 (IGF2BP2) and rs2736098 (*TERT*) as two independent risk factors for disease susceptibility (OR=1.36, CI 1.07-1.74; OR= 1.555, CI 1.182-2.041 respectively). In other words, the estimated risk of T2D for individuals with risk alleles in both genes was about three times higher than those of an individual without risk alleles.

**Table 3:** Final logistic regression model for T2D susceptibility. A stepwise regression procedure based on the AIC criterion was adopted to select the best set of variables.

**Variable**	**B**	**SE**	**Wald**	**P-value**	**OR (95% CI)**
Gender	-0.911	0.180	25.693	<0.001	0.402 (0.283-0.572)
BMI	0.058	0.019	9.015	0.003	1.059 (1.02-1.1)
Age	0.081	0.009	78.290	<0.001	1.084 (1.065-1.103)
Familiarity	2.564	0.189	184.020	<0.001	12.985 (8.966-18.807)
rs4402960-T	0.309	0.125	6.057	0.014	1.362 (1.065-1.741)
rs2736098-G	0.441	0.139	10.000	0.002	1.555 (1.182-2.041)
Constant	-6.548	0.826	62.888	<0.001	

Abbreviations: B, regression coefficient, SE, standard error; OR, odds ratio; CI, confidence interval.

**Figure 1:**
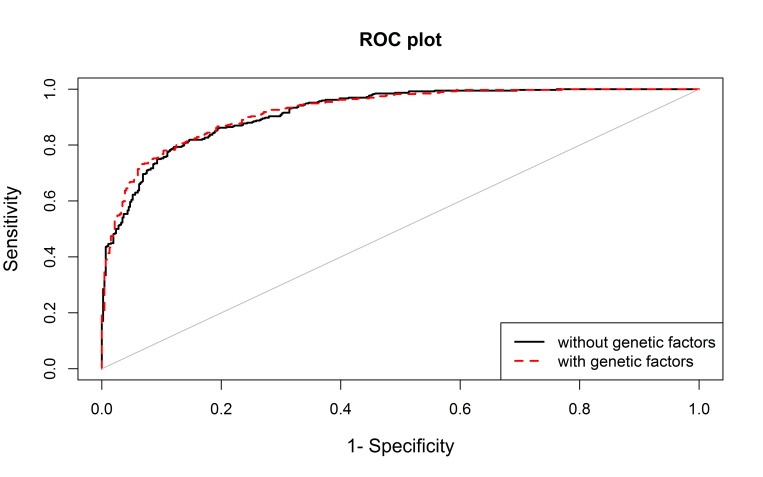
The area under the curve (AUC) for the model including non-genetic variables (age, sex, BMI and family history, in black) and for the model also including genetic data (rs4402960 and rs2736098, in red).

The performance of the model including the above variants was only slightly higher than the model including only non-genetic variables (area under the ROC curve (AUC) 0.923 vs 0.919; p-value=0.036) (Figure 1).

## SNPs association with diabetic complications

Table 4 shows demographics and clinical characteristics of T2D patients with and without micro- and macrovascular complications. Table S2 lists the results of the association test for each SNP, while Table 5 summarizes the most significant findings. The *UCP1* rs45539933-T allele resulted less frequent in patients with coexisting diabetic retinopathy compared to those without (OR 0.31, 95% CI 0.12-0.82; p=0.010). *UCP1* was also implicated in nephropathy risk, but in this case associations were found with rs3811787 and rs1800592, for which the minor alleles (G for both SNPs) acted as a protective factor against this diabetic complication (OR 0.55, 95% CI 0.33-0.98; p=0.031 for both SNPs). These two variants were in moderate LD (r^2^ =0.53).

**Table 4:** Microvascular and Macrovascular complications in Type 2 Diabetic patients.

	**Retinopathy**			**Nephropathy**			**Neuropathy**			**Ischemic heart disease and stroke**		
	Yes (n=111)	No (n=324)	P-value	Yes (n=54)	No (n=381)	P-value	Yes (n=76)	No (n=359)	P-value	Yes (n=76)	No (n=359)	P-value
Age (Mean, SD)	67.1 (7.86)	65.2 (7.87)	0.035	66.4 (9.06)	65.6 (7.73)	0.475	66.0 (6.73)	65.6 (8.13)	0.686	67.4 (6.4)	65.4 (8.1)	0.021
Sex (males, %)	65 (58.6)	181 (55.9)	0.621	39 (72.2)	207 (54.3)	0.013	56 (73.7)	190 (52.9)	0.001	52 (68.4)	194 54.0)	0.022
BMI, kg/m^2^ (Mean, SD)	27.8 (3.13)	29.0 (4.96)	0.002	29.2 (4.90)	28.6 (4.55)	0.396	29.0 (3.88)	28.7 (4.74)	0.626	28.7 (5.2)	28.7 (4.5)	0.903
Family history (n, %)	94 (84.7)	220 (68.3)	0.001	42 (77.8)	272 (71.8)	0.355	59 (77.6)	255 (71.4)	0.271	56 (73.7)	258 (72.3)	0.802
WHR (mean, SD)	0.93 (0.07)	0.93 (0.09)	0.984	0.95 (0.07)	0.93 (0.08)	0.127	0.95 (0.06)	0.93 (0.09)	0.036	0.96 (0.05)	0.93 (0.08)	0.183
HOMA-IR (mean, SD)	2.75 (2.38)	2.96 (2.93)	0.484	3.70 (3.18)	2.79 (2.72)	0.025	2.94 (2.25)	2.90 (2.90)	0.904	4.97 (4.53)	2.83 (2.69)	0.090
Fasting glucose, mg/dl (Mean, SD)	176.4 (50.88)	158.2 (45.44)	<0.001	182.7 (52.56)	160.0 (46.13)	0.004	179.6 (53.15)	159.3 (45.5)	0.002	170.7 (52.0)	161.4 (46.4)	0.140
Insulin, µIU/mL (mean, SD)	6.26 (5.02)	7.34 (5.48)	0.066	8.18 (6.55)	6.91 (5.19)	0.106	6.52 (4.12)	7.18 (5.61)	0.329	11.3 (9.7)	6.92 (5.1)	0.105
HbA1c, % (Mean, SD)	7.8 (1.11)	7.3 (1.30)	<0.001	7.9 (1.56)	7.4 (1.21)	0.027	7.8 (1.25)	7.4 (1.26)	0.002	7.6 (1.5)	7.4 (1.2)	0.199
Diabetes duration, years (Mean, SD)	21.3 (21.1)	11.2 (9.4)	<0.001	14.8 (12.2)	13.9 (10.9)	0.597	18.7 (11.9)	12.9 (10.6)	<0.001	15.8 (12.7)	13.6 (10.7)	0.134
Age at diagnosis (Mean, SD)	45.0 (11.9)	52.9 (10.6)	<0.001	50.5 (13.7)	50.8 (11.2)	0.882	46.3 (11.6)	51.8 (11.2)	<0.001	50.6 (12.7)	50.8 (11.2)	0.881

Abbreviations: SD, standard deviation; BMI, Body Mass Index; WHR, waist-to-hip ratio; HOMA-IR, homeostasis model assessment of insulin resistance; HbA1c, glycosylated haemoglobin.

**Table 5:** Association analysis of candidate SNPs with diabetic microvascular and macrovascular complications.

				**Retinopathy**		**Nephropathy**		**Ischemic heart disease and stroke**	
**Gene**	**SNP**	**Alleles**	**MAF**	**OR (95% CI)^a^**	**P-value**	**OR (95% CI)^a^**	**P-value**	**OR (95% CI)^a^**	**P-value**
ADIPOQ	rs266729	C/G	0.24	1.10 (0.74-1.63)	0.641	1.10 (0.70-1.73)	0.672	**0.61 (0.39-0.95)**	**0.024**
CAT	rs1001179	G/A	0.22	1.03 (0.66-1.63)	0.885	**0.48 (0.26-0.87)**	**0.010**	1.03 (0.66-1.6)	0.905
FTO	rs1121980	C/T	0.48	1.01 (0.70-1.44)	0.976	**0.64 (0.42-0.96)**	**0.030**	0.97 (0.68-1.38)	0.875
FTO	rs1421085	T/C	0.47	0.97 (0.68-1.38)	0.864	**0.63 (0.42-0.95)**	**0.026**	0.95 (0.67-1.35)	0.783
FTO	rs17817449	T/G	0.45	1.08 (0.76-1.53)	0.684	**0.66 (0.44-0.098)**	**0.044**	0.97 (0.69-1.37)	0.866
UCP1	rs1800592	A/G	0.24	0.84 (0.55-1.29)	0.426	**0.57 (0.33-0.98)**	**0.031**	1.10 (0.74-1.64)	0.643
UCP1	rs3811787	T/G	0.25	1.20 (0.80-1.79)	0.382	**0.57 (0.34-0.98)**	**0.031**	1.20 (0.81-1.77)	0.367
UCP1	rs45539933	C/T	0.06	**0.31 (0.12-0.82)**	**0.010**	0.52 (0.18-1.52)	0.198	1.08 (0.52-2.26)	0.837

Abbreviations: MAF, Minor Allele Frequency; OR, odds ratio; CI, confidence interval.

Significant p-values (<0.05) are highlighted in bold.

^a^ For each SNP an adjusted additive model was considered.

The minor alleles of three SNPs in the *FTO* gene (rs1121980-T, rs1421085-C and rs17817449-G), in LD with each other (r^2^>0.88), were instead associated with a significantly lower risk of nephropathy, with ORs 0.64 (95% CI 0.42-0.96; p=0.030), 0.63 (95% CI 0.42-0.95; p=0.026), and 0.66 (95% CI 0.44-0.98; p=0.044), respectively. The rs1001179-A allele in the *CAT* gene also displayed a similar association with nephropathy (OR 0.48, 95% CI 0.26-0.87; p=0.010).

As for macrovascular complications, we only found the *ADIPOQ* rs266729- G-allele associated with a decreased risk of macrovascular events (ischemic cardiovascular disease and stroke), with an OR of 0.61 per risk allele (95% CI 0.39-0.95; p=0.024).

## DISCUSSION

In this study, we show that SNP rs4402960 in the *IGF2BP2* gene, which encodes the Insulin-like growth factor 2 mRNA-binding protein 2, is significantly associated with an increased risk of T2D. Variants in *IGF2BP2* have been previously found to be significantly associated with alterations in insulin secretion and resistance [19], and *IGF2BP2* was found upregulated in the beta-cells of T2D patients [20]. Furthermore, a number of studies reported that the minor T allele of rs4402960 confers a higher risk of disease in different ethnic groups [21–23]. In agreement with these studies, we observed an additive genetic effect of this allelic variant on the disease risk. IGF2BP2 regulates the signalling activity of IGF2, which, in turn, controls growth, pancreatic development, and insulin signalling pathways [24]. In mice, IGF2BP2 can bind the mRNAs of genes encoding the mitochondrial uncoupling protein (*Ucp*1) and other mitochondrial components [25], strongly suggesting that IGF2BP2 might also confer disease susceptibility by affecting mitochondrial functions [26].

We also found a significant association between T2D and two variants (rs2735940 and rs2736098) in the *TERT* gene, which encodes for one of the major components of the enzyme telomerase that is essential for telomere maintenance. Previous studies suggested that telomere attrition may be a marker associated with the presence and the number of diabetic complications [27,28] and could provide additive prognostic information on mortality risk in T2D patients [29]. The rs2735940-C allele was previously associated with a lower transcriptional activity of the *TERT* promoter and decreased telomere length [30]. Contrasting results have also been reported regarding the association between rs2736098 variants and telomere length [30–33]. Here, we did not observe any correlation between the polymorphisms analysed and telomere length (data not shown), suggesting an independent effect on T2D risk. TERT exerts other extra-nuclear functions that might provide an explanation for the observed associations. For instance, it has been shown that oxidative stress induces the nuclear export of TERT and the increase of TERT in mitochondria [34]. Additionally, TERT regulates pathways involved in glucose uptake [35]. Taken together, these observations provide further support to the hypothesis of a link between mitochondria dysfunction and T2D [26].

Using a stepwise logistic regression, we confirmed rs4402960 (*IGF2BP2*) and rs2736098 (*TERT*) as two independent risk factors for T2D. Indeed, the simultaneous presence of both risk alleles confers a three-fold increased risk of developing the disease. The addition of these genetic variants into a model including conventional non-genetics risk factors provides a very small, but significant improvement in the ability to predict disease risk.

Microvascular (retinopathy, nephropathy and neuropathy), and macrovascular (ischemic heart disease and stroke) complications are common among people with diabetes and have profound effects on patients’ morbidity and mortality. Our observation of a higher prevalence of nephropathy, neuropathy, and ischemic heart disease and stroke among men was in general agreement with previous reports of gender differences in various diabetes-related comorbidities. In particular, men seem to be at higher risk for developing diabetic microvascular complications, while women have greater incidence of myocardial infarction and stroke mortality [36]. This may account for the observed higher percentage of men with diabetes outcomes.

Interesting findings emerged from the analysis of the effect of each selected variant on the risk of vascular complications. We found that the minor T allele of the missense variation rs45539933 in the *UCP1* reduces the risk of retinopathy. This result strongly supports the idea proposed by Kowluru and Mishra [37] that changes in mitochondrial function and increased oxidative stress are involved in the development of diabetic retinopathy. This notion is further bolstered by the finding that two other SNPs (rs1800592 and rs3811787) in the promoter region of *UCP1* gene are implicated in diabetic nephropathy risk. As an uncoupling protein, UCP1 acts in thermogenesis, regulation of energy expenditure, and in decreasing oxidative stress, which are processes deregulated in metabolic disorders [37]. In line with these observations, rs45539933 and rs1800592 are among the most commonly reported variants associated with obesity and diabetes [38], conditions that can either cause or worsen the progression of nephropathy.

The importance of obesity-related factors and oxidative stress in nephropathy risk is also supported by our finding that variants in the *FTO* and C*AT* genes, which encode the fat mass and obesity-associated and catalase proteins, respectively, are associated with this diabetic complication. Variations in the *FTO* intron 1, which affect primary transcript levels, have been widely related to obesity and obesity-related traits [39]. Some of the obesity-associated *FTO* SNPs, in particular rs9939609 and rs8050136, have also been associated with T2D. Although it is still controversial whether this association is fully mediated through the effect of FTO on BMI or not [40,41]. Bravard et al. [42] found increased FTO mRNA and protein levels in skeletal muscle from T2D patients and provided *in vitro* data supporting a potential implication of FTO in oxidative metabolism. Further evidence of this proposed relationship emanates from studies showing that FTO deficiency induces Ucp1 expression in the adipose tissue of mice [43]. In our study, none of the tested *FTO* SNPs were associated with T2D, but all affected the individual’s risk of nephropathy. For three of them (rs1421085, rs1121980 and rs17817449) the association was statistically significant (the minor alleles had a protective effect), while for two (rs9939609 and rs8050136) there was just a trend in the same direction. In line with our findings, rs8050136 has been recently reported to be associated with a lower estimated glomerular filtration rate in patients with T2D [44]. Of note, five of the *FTO* SNPs that we analysed are in LD with each other; therefore, these associations are not independent.

As for catalase, it is known that its deficiency is associated with increased intracellular stress which leads to insulin resistance and T2D [45]. Here, we report evidence that the minor A allele of the functional SNP rs1001179 has a protective effect with regard to developing nephropathy. The literature shows contrasting results about the association between this SNP and nephropathy [46], as well as about the functional effect of this allele. While some investigators reported higher catalase levels associated with the rs1001179-A allele [47], others found the opposite or no effect at all [48]. Our result supports the findings by Schults et al. [47] of an increased activity associated with this allele, which could be indicative of its greater antioxidant capacity.

*ADIPOQ* gene, encoding the adipocytes-derived protein adiponectin, was the only gene that we found to be associated with macrovascular complications. Due to its antioxidant, anti-inflammatory, and atheroprotective properties, adiponectin is considered as a useful cardiometabolic marker [49]. Low levels of this protein are predictive of coronary heart disease in several, but not all studies [50]. One of the SNPs associated with adiponectin level is the rs266729 C/G variant in the promoter region of the gene. We found the rs266729-G allele associated with a decreased risk of macrovascular outcomes. Consistent with a protective effect, homozygosity for this allele was found associated with increased antioxidant capacity and with a modest increase in the plasma levels of adiponectin in patients with diabetes [51]. However, there is inconsistency in published data about the exact effect of this allele on *ADIPOQ* expression as well as the genotype associated with the risk of cardiovascular diseases [50-52 and references therein]. Given that adiponectin has pleiotropic and, likely, independent effects, it is possible that the inconsistency depends on interactions with either other genetic loci or population-specific environmental risk factors that are not currently known. It is intriguing that also adiponectin was found to inhibit *Ucp1* gene expression in mice adipocytes [53].

In summary, we found SNPs in several genes of known importance to diabetes to be associated with T2D risk and its related vascular complications. These genes have distinct functions that, however, appear to converge on a common mechanism that is related to the regulation of mitochondrial-induced oxidative stress. This idea is suggested by the finding that variants in genes encoding for mitochondrial oxidative proteins, such as *UCP1* and *CAT,* are associated with the risk of diabetic complications. And, it is also supported by the observed associations with genes, such as *IGF2BP2*, *FTO* and *ADIPOQ*, whose products are likely to affect oxidative metabolism through the regulation of the expression of the *Ucp1*gene [25,43,53]. Our findings strongly suggest that more research is warranted to further explore the relationship between mitochondrial oxidative homeostasis, T2D, and individual predisposition to diabetic vascular complications.

## METHODS

## Study population

The cohort used in this study consists of 503 individuals (mean age 63. ±9.3) with T2D and 580 (mean age 62.1±10.9) unaffected individuals. Spouses of patients were enrolled as healthy control subjects. All subjects were collected by the Diabetoly Unit, INRCA (National Institute on Health and Science on Aging) in Ancona (Italy). To avoid population stratification effects, only individuals with at least two generations of ancestors from the Marche region (Central Italy) were included in this study. The study was approved by the Ethics Committee of INRCA, and written informed consent was obtained by all participants [18].

T2D was diagnosed according to the American Diabetes Association Criteria (American Diabetes Association, 2010). Criteria for patients' inclusion were: having body mass index (BMI) <40kg/m^2^, being between 35 and 85 years of age, being willing and able to provide written informed consent as well as to comply with the requirements of the study. Data on vital signs, anthropometric factors, medical history and behaviours as well as physical activity were collected. Additional data, including weight, height, BMI, waist circumference, hip circumference, glycaemia, glycated haemoglobin, insulin resistance and beta cell function in the form of homeostatic model assessment (HOMA) were obtained for each individual. Control subjects were carefully assessed and fully characterized using the same protocol used for T2D patients, in order to exclude the presence of T2D and of other diseases.

The presence/absence of diabetic complications was evidenced as follows: diabetic retinopathy by fundoscopy through dilated pupils and/or fluorescence angiography; incipient nephropathy, defined as an urinary albumin excretion rate >30 mg/24h and a normal creatinine clearance; renal failure, defined as an estimated glomerular filtration rate >60 mL/min per 1.73 m^2^; neuropathy established by electromyography; ischemic heart disease defined by clinical history, and/or ischemic electrocardiographic alterations; peripheral vascular disease including atherosclerosis obliterans and cerebrovascular disease on the basis of history, physical examinations and Doppler velocimetry.

Among T2D patients without complications, about a third were only on dietary restriction, whereas the remaining part of the patients without complications and those with complications were treated with Metformin and/or Sulfonylurea. About a third of T2D patients, especially those with long diabetes duration, were treated with insulin. T2D patients affected by cardiovascular diseases risk factors, including hypercholesterolemia and hypertension, were treated with statins, beta-blockers, calcium antagonists and ACE-inhibitors according to their clinical history.

## Measurements

Blood concentrations of fasting glucose, HbA1c, fasting insulin, were measured by standard procedures. Overnight fasting venous blood samples of all subjects were collected from 8:00 to 9:00 a.m. The samples were either analysed immediately or stored at -80°C for no more than 10 days. Measurements were taken in standard units. The BMI was calculated by dividing the weight in kilograms by height in meters. Glycaemia was measured in milliMoles/liter. HOMA assesses beta cell function and insulin sensitivity in percentages and by imputing plasma glucose levels and insulin levels into the model can derive the estimated insulin resistance.

## SNPs genotyping

Supplementary Table S1 reports the complete list of selected SNPs, their position, and known SNP-trait associations. DNA was extracted from whole blood (QIAmp 96 DNA Blood kit, QIAGEN). Genotyping analysis was performed by using SEQUENOM MassArray iPLEX technology, following the manufacturer's instructions. Genotype calls were analysed by using SEQUENOM Typer 4.0 software and the individual spectrograms were checked in order to evaluate the presence of calling errors.

## Quality control of genotype data

After genotype calling the dataset was subjected to a battery of QC tests. On the sample level, subjects with a proportion of missing genotypes higher than 10% were excluded from the study. On the SNP level, SNPs were excluded if they had a significant deviation from HWE (p<0.05) in the control group, a MAF lower than 5% and a MiF higher than 10%.

## Statistical analyses

Continuous and categorical variables were compared using the independent samples *t* test and the chi square test as appropriate. For each SNP, allele and genotype frequencies were estimated by gene counting from the observed genotypes. HWE was tested by Fisher’s exact test. The pairwise measures of LD between the analysed loci were carried out using Haploview.

## *Single-SNP analysis*

Logistic regression models were used to estimate how the variability of analysed genes influences the predisposition to T2D and related vascular complications. Age, gender, BMI, familiarity and duration of diabetes (when appropriate) were used as covariates in the formulated regression models. The OR was calculated coding genetic data in an additive fashion (number of risk alleles).

## *Genetic profiles associated with T2D*

To evaluate the combined effect of genetic variables on T2D susceptibility we used a stepwise procedure based on the Akaike information criterion (AIC) criterion to select the best set of variables. The performance of this model has been evaluated in terms of AUC. In order to evaluate how genetic variants can improve the ability to discriminate cases and controls we compared the performance of the model including only non-genetic variants (age, gender, BMI, and familiarity) with those also including the formulated genetic profile.

Considering that this is a study to confirm previous findings regarding known SNPs associated with T2D and its related vascular complications, a nominal threshold of 0.05 was set for statistical significance in all analyses.

Analyses have been performed using pROC, and SNPassoc packages of R (http://www.R-project.org/). The difference between AUC values was tested using the method proposed by DeLong [54].

## Supplementary Material

Supplementary File
