# Supplementary material for: Genes associated with Type 2 Diabetes and vascular complications
Source: Aging (Albany NY). 2018 Feb 4;10(2):178–96. doi: 10.18632/aging.101375 (PMC5842840; doi:10.18632/aging.101375)
Supplement: Supplementary File [file aging-10-101375-s001.pdf]

SUPPLEMENTARY MATERIAL

Table S1. Basic characteristics of 40 genotyped SNPs in candidate genes and their associated traits.

Gene	SNP	CHR	Mutation Type	Known associations of interest	References
ADIPOQ	rs266729	3	Upstream gene variant	Nephropathy and Retinopathy in T2D	Cai et al. 2015; Li et al. 2015
ADIPOQ, ADIPOQ-AS1	rs1063539	3	3'UTR variant, Upstream gene variant	Diabetes mellitus and Hypertension	Tong et al. 2013; Jiang et al. 2014
APOE	rs440446	19	Missense variant	Not associated to T2D	Geng et al. 2011
CAT	rs1001179	11	Upstream gene variant	Oxidative stress, Type 2 Diabetes	Pacal et al. 2011
CLPTM1L	rs401681	5	Upstream variant 2KB	Pancreatic cancer, T2D	Wu et al. 2015
DDAH1	rs669173	1	Intron variant, Non coding transcript variant, Downstream gene variant	ADMA(Asymmetric dimethylarginine) levels	Abhary et al. 2010
DDAH1	rs13373844	1	Intron variant, Non coding transcript variant	ADMA(Asymmetric dimethylarginine) levels	Abhary et al. 2010
DDAH1	rs7521189	1	Intron variant, Non coding transcript variant	ADMA(Asymmetric dimethylarginine) levels	Abhary et al. 2010
EPO	rs1617640	7	Upstream variant 2KB	Diabetic Retinopathy, Diabetic Nephropathy	Tong et al. 2008
EPO	rs507392	7	Intron variant	Diabetic Retinopathy, Diabetic Nephropathy	Tong et al. 2008
EPO	rs551238	7	Downstream gene variant 500B	Diabetic Retinopathy, Diabetic Nephropathy	Tong et al. 2008
FTO	rs1121980	16	Intron variant, NMD transcript variant, Non coding transcript variant	Susceptibility to Type 2 Diabetes, Obesity	Li et al. 2011
FTO	rs1421085	16	Intron variant, NMD transcript variant, Non coding transcript variant	BMI and Type 2 Diabetes	Akiyama et al. 2014
FTO	rs17817449	16	Intron variant, NMD transcript variant, Non coding transcript variant	Susceptibility to Type 2 Diabetes, Obesity	Basile et al. 2014
FTO	rs8047395	16	Intron variant, NMD transcript variant, Non coding transcript variant	Susceptibility to Type 2 Diabetes, Obesity	Akiyama et al. 2014
FTO	rs8050136	16	Intron variant, NMD transcript variant, Non coding transcript variant	Susceptibility to Type 2 Diabetes	Chang et al. 2014
FTO	rs9939609	16	Intron variant, NMD transcript variant, Non coding transcript variant	BMI and Type 2 Diabetes	Akiyama et al. 2014

HIF1A	rs11549465	14	Missense variant	Susceptibility to Type 1 and Type 2 Diabetes	Nagy et al. 2009
IGF2BP2	rs1470579	3	Intron variant	Susceptibility to Type 2 Diabetes	Park et al. 2015
IGF2BP2	rs4402960	3	Intron variant	Susceptibility to Type 2 Diabetes	Scott et al. 2007; The Wellcome Trust Case Control 2007; Zeggini et al. 2007
IRS1	rs2943641	2	Intergenic variant (500 kb upstream)	Insulin resistance and Hyperinsulinemia	Rung et al. 2009
KCNJ11	rs5215	11	Missense variant	Susceptibility to Type 2 Diabetes	Zeggini et al. 2008
KCNQ1	rs2237892	11	Intron variant	Susceptibility to Type 2 Diabetes	Kong et al. 2009
SLC30A8	rs13266634	8	Missense variant	Susceptibility to Type 2 Diabetes	Sladek et al. 2007
SLC30A8	rs16889462	8	Missense variant	Susceptibility to Type 2 Diabetes	Labruna et al. 2011; Sun et al. 2014
SOD2	rs4880	6	Missense variant	Diabetic Nephropathy	Mollsten et al. 2007
TCF7L2	rs7901695	10	Intron variant	Susceptibility to Type 2 Diabetes	Scott et al. 2007; The Wellcome Trust Case Control 2007; Zeggini et al. 2007
TCF7L2	rs7903146	10	Intron variant	Susceptibility to Type 2 Diabetes	Grant et al. 2006
TERC	rs12696304	3	Downstream gene variant (1.5 kb)	Human Leukocyte Telomere Length and the Risk of T2D	Codd et al. 2010; Al Khaldi et al. 2015
TERT	rs2735940	5	Upstream variant 2KB	Susceptibility to Breast cancer	Pellatt et al. 2013
TERT	rs2736098	5	Synonymous variant	Cancer, T2D, leukocyte telomere length	You et al. 2012, Wu et al. 2015
TERT	rs2736109	5	Upstream variant 2KB	Telomere lengths, Susceptibility to Breast and Ovarian cancer	Bojesen et al. 2013
TERT	rs2853669	5	Upstream variant 2KB	Susceptibility to Type 2 Diabetes	You et al. 2012,
UCP1	rs1800592	4	Upstream gene variant	Abdominal Fat, Blood pressure, Retinopathy, Insulin resistance, T2D	Fukuyama et al. 2006; Cha et al. 2008; Ho Cha et al. 2008; Zhang et al. 2015
UCP1	rs3811787	4	Upstream gene variant	Abdominal Fat, Obesity	Ho Cha et al. 2008, Labruna et al. 2011

UCP1	rs3811791	4	Upstream gene variant	Susceptibility to Type 2 Diabetes	Lee et al. 2008
UCP1	rs45539933	4	Missense variant	Blood pressure, Obesity	Cha et al. 2008, Labruna et al. 2011
UCP2	rs660339	11	Missense variant	Diabetic Retinopathy	Crispim et al. 2010
UCP3	rs1800849	11	5'UTR variant	Age, Type 2 Diabetes, Abdominal Obesity	Pinelli et al. 2006
VEGFA	rs3025021	6	Intron variant, Non coding transcript variant, Non coding transcript exon variant, downstream gene variant	Diabetic Retinopathy	Abhary et al. 2010,

REFERENCES

1. Abhary S, Burdon KP, Casson RJ, Goggin M, Petrovsky NP, Craig JE. Association between erythropoietin gene polymorphisms and diabetic retinopathy. Arch Ophthalmol. 2010; 128:102–06. https://doi.org/10.1001/archophthalmol.2009.355

2. Abhary S, Burdon KP, Kuot A, Javadiyan S, Whiting MJ, Kasmeridis N, Petrovsky N, Craig JE. Sequence variation in *DDAH1* and *DDAH2* genes is strongly and additively associated with serum ADMA concentrations in individuals with type 2 diabetes. PLoS One. 2010; 5:e9462. https://doi.org/10.1371/journal.pone.0009462

3. Abhary S, Burdon KP, Kuot A, Javadiyan S, Whiting MJ, Kasmeridis N, Petrovsky N, Craig JE. Sequence variation in DDAH1 and DDAH2 genes is strongly and additively associated with serum ADMA concentrations in individuals with type 2 diabetes. PLoS One. 2010; 5:e9462. https://doi.org/10.1371/journal.pone.0009462

4. Akiyama K, Takeuchi F, Isono M, Chakrawarthy S, Nguyen QN, Wen W, Yamamoto K, Katsuya T, Kasturiratne A, Pham ST, Zheng W, Matsushita Y, Kishimoto M, et al. Systematic fine-mapping of association with BMI and type 2 diabetes at the FTO locus by integrating results from multiple ethnic groups. PLoS One. 2014; 9:e101329. https://doi.org/10.1371/journal.pone.0101329

5. Al Khaldi R, Mojiminiyi O, AlMulla F, Abdella N. Associations of TERC Single Nucleotide Polymorphisms with Human Leukocyte Telomere Length and the Risk of Type 2 Diabetes Mellitus. PLoS One. 2015; 10:e0145721. https://doi.org/10.1371/journal.pone.0145721

6. Basile KJ, Johnson ME, Xia Q, Grant SF. Genetic susceptibility to type 2 diabetes and obesity: follow-up of findings from genome-wide association studies. Int J Endocrinol. 2014; 2014:769671. https://doi.org/10.1155/2014/769671

7. Bojesen SE, Pooley KA, Johnatty SE, Beesley J, Michailidou K, Tyrer JP, Edwards SL, Pickett HA, Shen HC, Smart CE, Hillman KM, Mai PL, Lawrenson K, Stutz MD, et al. Multiple independent variants at the TERT locus are associated with telomere length and risks of breast and ovarian cancer. Nature genetics. 2013; 45: 371-384e372.

8. Cai Y, Zeng T, Chen L. Association of adiponectin polymorphisms with the risk of diabetic nephropathy in type 2 diabetes: a meta-analysis. J Diabetes. 2015; 7:31–40. https://doi.org/10.1111/1753-0407.12166

9. Cha MH, Kang BK, Suh D, Kim KS, Yang Y, Yoon Y. Association of UCP1 genetic polymorphisms with blood pressure among Korean female subjects. J Korean Med Sci. 2008; 23:776–80. https://doi.org/10.3346/jkms.2008.23.5.776

10. Chang YC, Liu PH, Yu YH, Kuo SS, Chang TJ, Jiang YD, Nong JY, Hwang JJ, Chuang LM. Validation of type 2 diabetes risk variants identified by genome-wide association studies in Han Chinese population: a replication study and meta-analysis. PLoS One. 2014; 9:e95045. https://doi.org/10.1371/journal.pone.0095045

11. Codd V, Mangino M, van der Harst P, Braund PS, Kaiser M, Beveridge AJ, Rafelt S, Moore J, Nelson C,

Soranzo N, Zhai G, Valdes AM, Blackburn H, et al, and Wellcome Trust Case Control Consortium. Common variants near TERC are associated with mean telomere length. Nat Genet. 2010; 42:197–99. https://doi.org/10.1038/ng.532

12. Crispim D, Fagundes NJ, dos Santos KG, Rheinheimer J, Bouças AP, de Souza BM, Macedo GS, Leiria LB, Gross JL, Canani LH. Polymorphisms of the UCP2 gene are associated with proliferative diabetic retinopathy in patients with diabetes mellitus. Clin Endocrinol (Oxf). 2010; 72:612–19. https://doi.org/10.1111/j.1365-2265.2009.03684.x

13. Fukuyama K, Ohara T, Hirota Y, Maeda K, Kuno S, Zenibayashi M, Teranishi T, Kouyama K, Maeda E, Sakamoto N, Kasuga M. Association of the -112A>C polymorphism of the uncoupling protein 1 gene with insulin resistance in Japanese individuals with type 2 diabetes. Biochem Biophys Res Commun. 2006; 339:1212–16. https://doi.org/10.1016/j.bbrc.2005.11.140

14. Geng H, Law PP, Ng MC, Li T, Liang LY, Ge TF, Wong KB, Liang C, Ma RC, So WY, Chan JC, Ho YY. APOE genotype-function relationship: evidence of -491 A/T promoter polymorphism modifying transcription control but not type 2 diabetes risk. PLoS One. 2011; 6:e24669. https://doi.org/10.1371/journal.pone.0024669

15. Grant SF, Thorleifsson G, Reynisdottir I, Benediktsson R, Manolescu A, Sainz J, Helgason A, Stefansson H, Emilsson V, Helgadottir A, Styrkarsdottir U, Magnusson KP, Walters GB, et al. Variant of transcription factor 7-like 2 (TCF7L2) gene confers risk of type 2 diabetes. Nat Genet. 2006; 38:320–23. https://doi.org/10.1038/ng1732

16. Cha MH, Kim KS, Suh D, Chung SI, Yoon Y. A UCP1-412A>C polymorphism is associated with abdominal fat area in Korean women. Hereditas. 2008; 145:231–37. https://doi.org/10.1111/j.1601-5223.2008.02071.x

17. Jiang B, Liu Y, Liu Y, Fang F, Wang X, Li B. Association of four insulin resistance genes with type 2 diabetes mellitus and hypertension in the Chinese Han population. Mol Biol Rep. 2014; 41:925–33. https://doi.org/10.1007/s11033-013-2937-0

18. Kong A, Steinthorsdottir V, Masson G, Thorleifsson G, Sulem P, Besenbacher S, Jonasdottir A, Sigurdsson A, Kristinsson KT, Jonasdottir A, Frigge ML, Gylfason A, Olason PI, et al, and DIAGRAM Consortium. Parental origin of sequence variants associated with complex diseases. Nature. 2009; 462:868–74. https://doi.org/10.1038/nature08625

19. Labruna G, Pasanisi F, Fortunato G, Nardelli C, Finelli C, Farinaro E, Contaldo F, Sacchetti L. Sequence Analysis of the UCP1 Gene in a Severe Obese Population from Southern Italy. J Obes. 2011; 2011:269043. https://doi.org/10.1155/2011/269043

20. Lee HJ, Ryu HJ, Shin HD, Park BL, Kim JY, Cho YM, Park KS, Song J, Oh B. Associations between polymorphisms in the mitochondrial uncoupling proteins (UCPs) with T2DM. Clin Chim Acta. 2008; 398:27–33. https://doi.org/10.1016/j.cca.2008.07.029

21. Li S, Zhao JH, Luan J, Langenberg C, Luben RN, Khaw KT, Wareham NJ, Loos RJ. Genetic predisposition to obesity leads to increased risk of type 2 diabetes. Diabetologia. 2011; 54:776–82. https://doi.org/10.1007/s00125-011-2044-5

22. Li Y, Wu QH, Jiao ML, Fan XH, Hu Q, Hao YH, Liu RH, Zhang W, Cui Y, Han LY. Gene-environment interaction between adiponectin gene polymorphisms and environmental factors on the risk of diabetic retinopathy. J Diabetes Investig. 2015; 6:56–66. https://doi.org/10.1111/jdi.12249

23. Möllsten A, Marklund SL, Wessman M, Svensson M, Forsblom C, Parkkonen M, Brismar K, Groop PH, Dahlquist G. A functional polymorphism in the manganese superoxide dismutase gene and diabetic nephropathy. Diabetes. 2007; 56:265–69. https://doi.org/10.2337/db06-0698

24. Nagy G, Kovacs-Nagy R, Kereszturi E, Somogyi A, Szekely A, Nemeth N, Hosszufalusi N, Panczel P, Ronai Z, Sasvari-Szekely M. Association of hypoxia inducible factor-1 alpha gene polymorphism with both type 1 and type 2 diabetes in a Caucasian (Hungarian) sample. BMC Med Genet. 2009; 10:79. https://doi.org/10.1186/1471-2350-10-79

25. Pácal L, Varvařovská J, Rušavý Z, Lacigová S, Stětina R, Racek J, Pomahačová R, Tanhäuserová V, Kaňková K. Parameters of oxidative stress, DNA damage and DNA repair in type 1 and type 2 diabetes mellitus. Arch Physiol Biochem. 2011; 117:222–30. https://doi.org/10.3109/13813455.2010.551135

26. Park HY, Choi HJ, Hong YC. Utilizing Genetic Predisposition Score in Predicting Risk of Type 2 Diabetes Mellitus Incidence: A Community-based Cohort Study on Middle-aged Koreans. J Korean Med Sci. 2015; 30:1101–09. https://doi.org/10.3346/jkms.2015.30.8.1101

27. Pellatt AJ, Wolff RK, Torres-Mejia G, John EM, Herrick JS, Lundgreen A, Baumgartner KB, Giuliano AR, Hines LM, Fejerman L, Cawthon R, Slattery ML. Telomere length, telomere-related genes, and breast cancer risk: the breast cancer health disparities study. Genes Chromosomes Cancer. 2013; 52:595–609.

https://doi.org/10.1002/gcc.22056

28. Pinelli M, Giacchetti M, Acquaviva F, Cocozza S, Donnarumma G, Lapice E, Riccardi G, Romano G, Vaccaro O, Monticelli A. Beta2-adrenergic receptor and UCP3 variants modulate the relationship between age and type 2 diabetes mellitus. BMC Med Genet. 2006; 7:85. https://doi.org/10.1186/1471-2350-7-85

29. Rung J, Cauchi S, Albrechtsen A, Shen L, Rocheleau G, Cavalcanti-Proença C, Bacot F, Balkau B, Belisle A, Borch-Johnsen K, Charpentier G, Dina C, Durand E, et al. Genetic variant near IRS1 is associated with type 2 diabetes, insulin resistance and hyperinsulinemia. Nat Genet. 2009; 41:1110–15. https://doi.org/10.1038/ng.443

30. Scott LJ, Mohlke KL, Bonnycastle LL, Willer CJ, Li Y, Duren WL, Erdos MR, Stringham HM, Chines PS, Jackson AU, Prokunina-Olsson L, Ding CJ, Swift AJ, et al. A genome-wide association study of type 2 diabetes in Finns detects multiple susceptibility variants. Science. 2007; 316:1341–45. https://doi.org/10.1126/science.1142382

31. Sladek R, Rocheleau G, Rung J, Dina C, Shen L, Serre D, Boutin P, Vincent D, Belisle A, Hadjadj S, Balkau B, Heude B, Charpentier G, et al. A genome-wide association study identifies novel risk loci for type 2 diabetes. Nature. 2007; 445:881–85. https://doi.org/10.1038/nature05616

32. Sun X, Yu W, Hu C. Genetics of type 2 diabetes: insights into the pathogenesis and its clinical application. BioMed Res Int. 2014; 2014:926713. https://doi.org/10.1155/2014/926713

33. Wellcome Trust Case Control Consortium. Genome-wide association study of 14,000 cases of seven common diseases and 3,000 shared controls. Nature. 2007; 447:661–78. https://doi.org/10.1038/nature05911

34. Tong Z, Yang Z, Patel S, Chen H, Gibbs D, Yang X, Hau VS, Kaminoh Y, Harmon J, Pearson E, Buehler J, Chen Y, Yu B, et al. D. Genetics of, G. Diabetic Complication Study, L. B. Jorde, D. Y. Li, L. P. Aiello, M. R. Pollak and K. Zhang. Promoter polymorphism of the erythropoietin gene in severe diabetic eye and kidney complications. Proceedings of the National Academy of Sciences of the United States of America. 2008. 105: 6998-7003.

35. Tong G, Wang N, Leng J, Tong X, Shen Y, Yang J, Ye X, Zhou L, Zhou Y. Common variants in adiponectin gene are associated with coronary artery disease and angiographical severity of coronary atherosclerosis in type 2 diabetes. Cardiovasc Diabetol. 2013; 12:67–67. https://doi.org/10.1186/1475-2840-12-67

36. Wu L, Rabe KG, Petersen GM. Do variants associated with susceptibility to pancreatic cancer and type 2 diabetes reciprocally affect risk? PLoS One. 2015; 10:e0117230. https://doi.org/10.1371/journal.pone.0117230

37. You NC, Chen BH, Song Y, Lu X, Chen Y, Manson JE, Kang M, Howard BV, Margolis KL, Curb JD, Phillips LS, Stefanick ML, Tinker LF, Liu S. A prospective study of leukocyte telomere length and risk of type 2 diabetes in postmenopausal women. Diabetes. 2012; 61:2998–3004. https://doi.org/10.2337/db12-0241

38. Zeggini E, Weedon MN, Lindgren CM, Frayling TM, Elliott KS, Lango H, Timpson NJ, Perry JR, Rayner NW, Freathy RM, Barrett JC, Shields B, Morris AP, et al, and Wellcome Trust Case Control Consortium (WTCCC). Replication of genome-wide association signals in UK samples reveals risk loci for type 2 diabetes. Science. 2007; 316:1336–41. https://doi.org/10.1126/science.1142364

39. Zeggini E, Scott LJ, Saxena R, Voight BF, Marchini JL, Hu T, de Bakker PI, Abecasis GR, Almgren P, Andersen G, Ardlie K, Boström KB, Bergman RN, et al, and Wellcome Trust Case Control Consortium. Meta-analysis of genome-wide association data and large-scale replication identifies additional susceptibility loci for type 2 diabetes. Nat Genet. 2008; 40:638–45. https://doi.org/10.1038/ng.120

40. Zhang Y, Meng N, Lv Z, Li H, Qu Y. The gene polymorphisms of UCP1 but not PPAR γ and TCF7L2 are associated with diabetic retinopathy in Chinese type 2 diabetes mellitus cases. Acta Ophthalmol. 2015; 93:e223–29. https://doi.org/10.1111/aos.12542

Table S2. Association analysis of candidate SNPs with diabetic microvascular and macrovascular complications.

				Retinopathy		Nephropathy		Neuropathy		Ischemic heart disease and stroke	
Gene	SNP	Alleles	MAF	OR (95% CI)[a]	P-value	OR (95% CI)[a]	P-value	OR (95% CI)[a]	P-value	OR (95% CI)a	P-value
ADIPOQ	rs266729	C/G	0.24	1.10 (0.74-1.63)	0.641	1.1 (0.70-1.73)	0.672	0.9 (0.58-1.38)	0.616	0.61 (0.39-0.95)	0.024
ADIPOQ-AS1	rs1063539	G/C	0.10	0.60 (0.30-1.22)	0.143	0.91 (0.43-1.90)	0.795	0.46 (0.19-1.12)	0.065	0.83 (0.43-1.64)	0.592
APOE	rs440446	G/C	0.39	1.13 (0.79-1.62)	0.510	1.12 (0.73-1.71)	0.617	1.06 (0.71-1.59)	0.760	1.16 (0.81-1.67)	0.425
CAT	rs1001179	G/A	0.22	1.03 (0.66-1.63)	0.885	0.48 (0.26-0.87)	0.010	0.93 (0.56-1.52)	0.760	1.03 (0.66-1.6)	0.905
CLPTM1L	rs401681	C/T	0.40	1.10 (0.76-1.58)	0.627	1.33 (0.86-2.04)	0.197	0.95 (0.64-1.41)	0.792	0.95 (0.65-1.38)	0.794
DDAH1	rs13373844	A/C	0.35	1.19 (0.82-1.71)	0.366	0.88 (0.57-1.35)	0.547	1.13 (0.77-1.67)	0.533	0.92 (0.63-1.33)	0.654
DDAH1	rs7521189	A/G	0.46	1.09 (0.76-1.57)	0.624	0.95 (0.62-1.44)	0.800	1.18 (0.8-1.75)	0.403	1.09 (0.76-1.56)	0.641
EPO	rs1617640	T/G	0.32	0.95 (0.65-1.39)	0.804	0.89 (0.57-1.37)	0.585	1.36 (0.92-2.02)	0.128	1.01 (0.70-1.47)	0.948
EPO	rs507392	T/C	0.32	0.92 (0.62-1.35)	0.665	0.92 (0.59-1.43)	0.713	1.38 (0.93-2.06)	0.114	1.08 (0.73-1.58)	0.709
EPO	rs551238	A/C	0.32	0.95 (0.65-1.39)	0.804	0.89 (0.57-1.37)	0.585	1.36 (0.92-2.02)	0.128	1.01 (0.70-1.47)	0.948
FTO	rs1121980	C/T	0.48	1.01 (0.70-1.44)	0.976	0.64 (0.42-0.96)	0.030	1.15 (0.79-1.67)	0.461	0.97 (0.68-1.38)	0.875
FTO	rs1421085	T/C	0.47	0.97 (0.68-1.38)	0.864	0.63 (0.42-0.95)	0.026	1.25 (0.86-1.82)	0.240	0.95 (0.67-1.35)	0.783
FTO	rs17817449	T/G	0.45	1.08 (0.76-1.53)	0.684	0.66 (0.44-0.98)	0.044	1.07 (0.74-1.55)	0.704	0.97 (0.69-1.37)	0.866
FTO	rs8050136	C/A	0.45	1.10 (0.77-1.57)	0.594	0.68 (0.45-1.03)	0.068	1.11 (0.76-1.61)	0.590	0.98 (0.69-1.39)	0.907
FTO	rs9939609	T/A	0.45	1.06 (0.74-1.52)	0.739	0.67 (0.45-1.02)	0.057	1.09 (0.75-1.58)	0.644	0.99 (0.70-1.41)	0.974
HIF1A	rs11549465	C/T	0.15	1.24 (0.75-2.05)	0.398	0.95 (0.53-1.71)	0.860	0.9 (0.52-1.57)	0.708	1.50 (0.93-2.41)	0.101
IGF2BP2	rs4402960	G/T	0.37	1.32 (0.92-1.89)	0.133	1.04 (0.68-1.59)	0.854	1.10 (0.74-1.64)	0.63	1.23 (0.86-1.77)	0.262
IRS1 (500 kb downstream)	rs2943641	C/T	0.40	1.22 (0.86-1.75)	0.267	1.03 (0.69-1.56)	0.871	0.71 (0.48-1.06)	0.086	0.93 (0.65-1.34)	0.712
KCNJ11	rs5215	T/C	0.34	1.14 (0.79-1.63)	0.483	1.17 (0.77-1.77)	0.463	0.78 (0.52-1.16)	0.209	0.89 (0.61-1.29)	0.527
KCNQ1	rs2237892	C/T	0.05	0.80 (0.33-1.91)	0.603	1.03 (0.41-2.57)	0.951	2.06 (0.95-4.45)	0.076	0.70 (0.29-1.73)	0.429
TCF7L2	rs7903146	C/T	0.44	0.70 (0.47-1.03)	0.070	1.21 (0.79-1.86)	0.373	0.84 (0.55-1.27)	0.399	0.97 (0.67-1.40)	0.866
TERC	rs12696304	C/G	0.27	0.86 (0.58-1.25)	0.420	1.20 (0.78-1.84)	0.410	0.72 (0.47-1.11)	0.125	0.89 (0.61-1.32)	0.573
TERT	rs2735940	T/C	0.42	1.14 (0.78-1.67)	0.498	1.22 (0.79-1.87)	0.374	0.72 (0.47-1.09)	0.116	1.28 (0.88-1.87)	0.191
TERT	rs2736098	G/A	0.29	1.01 (0.65-1.56)	0.973	0.69 (0.41-1.16)	0.151	1.48 (0.92-2.38)	0.105	1.08 (0.70-1.67)	0.726
TERT	rs2736109	G/A	0.41	1.04 (0.71-1.51)	0.844	0.69 (0.44-1.09)	0.108	0.94 (0.63-1.42)	0.781	1.07 (0.73-1.57)	0.726
UCP1	rs1800592	A/G	0.24	0.84 (0.55-1.29)	0.426	0.57 (0.33-0.98)	0.031	0.86 (0.54-1.36)	0.509	1.10 (0.74-1.64)	0.643

UCP1	rs3811787	T/G	0.25	1.20 (0.80-1.79)	0.382	0.57 (0.34-0.98)	0.031	1.02 (0.66-1.57)	0.945	1.20 (0.81-1.77)	0.367
UCP1	rs45539933	C/T	0.06	0.31 (0.12-0.82)	0.010	0.52 (0.18-1.52)	0.198	1.46 (0.68-3.13)	0.340	1.08 (0.52-2.26)	0.837
UCP2	rs660339	C/T	0.34	0.79 (0.54-1.15)	0.211	1.43 (0.93-2.18)	0.102	1.03 (0.69-1.55)	0.882	0.91 (0.62-1.34)	0.635
UCP3	rs1800849	C/T	0.14	0.88 (0.52-1.49)	0.627	1.72 (0.99-2.99)	0.060	1.13 (0.65-1.97)	0.660	0.86 (0.50-1.48)	0.586
VEGFA	rs3025021	C/T	0.32	1.04 (0.70-1.56)	0.841	1.03 (0.64-1.65)	0.894	0.98 (0.63-1.51)	0.916	0.79 (0.52-1.21)	0.278

Abbreviation: MAF, Minor Allele Frequency; OR, odds ratio; CI, confidence interval.
Significant p-values (<0.10) are highlighted in bold.
[a] At each SNP, an additive model was considered. Age. sex. BMI and familiarity were included as covariates.
